# Glutathione: A Samsonian life-sustaining small molecule that protects against oxidative stress, ageing and damaging inflammation

**DOI:** 10.3389/fnut.2022.1007816

**Published:** 2022-11-01

**Authors:** Carlos A. Labarrere, Ghassan S. Kassab

**Affiliations:** California Medical Innovations Institute, San Diego, CA, United States

**Keywords:** glutathione, oxidative stress, reactive oxygen species, nuclear factor erythroid 2-related factor 2, inflammaging, chronic obstructive pulmonary disease, atherosclerosis, COVID-19

## Abstract

Many local and systemic diseases especially diseases that are leading causes of death globally like chronic obstructive pulmonary disease, atherosclerosis with ischemic heart disease and stroke, cancer and severe acute respiratory syndrome coronavirus 2 (SARS-CoV-2) causing coronavirus disease 19 (COVID-19), involve both, (1) oxidative stress with excessive production of reactive oxygen species (ROS) that lower glutathione (GSH) levels, and (2) inflammation. The GSH tripeptide (γ- L-glutamyl-L-cysteinyl-glycine), the most abundant water-soluble non-protein thiol in the cell (1–10 mM) is fundamental for life by (a) sustaining the adequate redox cell signaling needed to maintain physiologic levels of oxidative stress fundamental to control life processes, and (b) limiting excessive oxidative stress that causes cell and tissue damage. GSH activity is facilitated by activation of the Kelch-like ECH-associated protein 1 (Keap1)-Nuclear factor erythroid 2-related factor 2 (Nrf2)-antioxidant response element (ARE) redox regulator pathway, releasing Nrf2 that regulates expression of genes controlling antioxidant, inflammatory and immune system responses. GSH exists in the thiol-reduced (>98% of total GSH) and disulfide-oxidized (GSSG) forms, and the concentrations of GSH and GSSG and their molar ratio are indicators of the functionality of the cell. GSH depletion may play a central role in inflammatory diseases and COVID-19 pathophysiology, host immune response and disease severity and mortality. Therapies enhancing GSH could become a cornerstone to reduce severity and fatal outcomes of inflammatory diseases and COVID-19 and increasing GSH levels may prevent and subdue these diseases. The life value of GSH makes for a paramount research field in biology and medicine and may be key against systemic inflammation and SARS-CoV-2 infection and COVID-19 disease. In this review, we emphasize on (1) GSH depletion as a fundamental risk factor for diseases like chronic obstructive pulmonary disease and atherosclerosis (ischemic heart disease and stroke), (2) importance of oxidative stress and antioxidants in SARS-CoV-2 infection and COVID-19 disease, (3) significance of GSH to counteract persistent damaging inflammation, inflammaging and early (premature) inflammaging associated with cell and tissue damage caused by excessive oxidative stress and lack of adequate antioxidant defenses in younger individuals, and (4) new therapies that include antioxidant defenses restoration.

## Introduction

Glutathione (GSH) is a unique molecule essential for life that participates in key aspects of cellular homeostasis, having a paramount role in defense against the oxidative damage that occurs during all different diseases including coronavirus disease 19 (COVID-19) disease. GSH has a central participation in *trans-*hydrogenation reactions needed to maintain a reduced state of sulfhydryl groups of other molecules, proteins and enzymes, as well as formation of deoxyribonucleotides and vitamin reduction (1–5). GSH has the function of “master antioxidant” in all tissues and is involved in antioxidant defense, detoxication of xenobiotics, intracellular redox homeostasis, cysteine carrier/storage, cell signaling, protein folding and function, gene expression, cell differentiation/proliferation, immune response and antiviral defense, that make it a “Samsonian (mighty) little molecule” (1, 2). The high (millimolar) concentration of the reduced form highlights its central role in the control of those processes (5–8). The central role of GSH in oxidative stress and inflammation, in the pathophysiology of inflammatory diseases and COVID-19, and in host immune response and disease severity and mortality, makes GSH a little but powerful player in maintaining health and avoiding disease. In this review we will focus on (a) GSH depletion as a fundamental risk factor for diseases like chronic obstructive pulmonary disease and atherosclerosis (ischemic heart disease and stroke), (b) importance of oxidative stress and antioxidants in severe acute respiratory syndrome coronavirus 2 (SARS-CoV-2) infection and COVID-19 disease, (c) significance of GSH to counteract persistent damaging inflammation, inflammaging and early (premature) inflammaging associated with cell and tissue damage caused by excessive oxidative stress and lack of adequate antioxidant defenses in younger individuals, and (d) new therapies that include antioxidant defenses restoration.

### Glutathione brief history

Glutathione was discovered in 1888 by de Rey-Pailhade and initially named “philothion” (from the Greek words meaning “love” and “sulfur”) because of its reactivity with sulfur to form hydrogen sulfide (4, 9). Subsequently, Hopkins reported this substance as a dipeptide containing glutamate and cysteine, and he named it “glutathione” (10), which is a tripeptide consisting of glutamate, cysteine, and glycine (11, 12). Harington and Mead finally described the correct chemical structure of the tripeptide in 1935 (13). GSH was virtually forgotten for 40 years until in 1969, Kosower and Kosower (14) emphasized the scant GSH research in those days. GSH research had a great momentum especially in the 1980s, with studies carried out by Meister and his collaborators who contributed to understanding the physiological functions and the metabolism (4).

### Glutathione composition and synthesis

**FIGURE 1:**
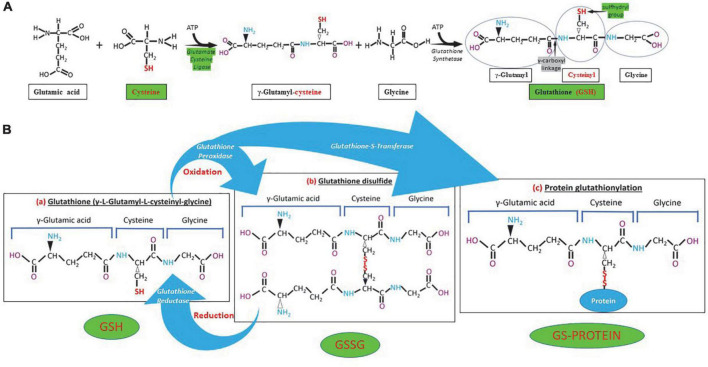
Glutathione (GSH) synthesis, chemical structure and different forms of GSH. **(A)** GSH is synthesized in the cytosol in two steps. The first step is the formation of γ-glutamylcysteine from glutamate and cysteine by the enzyme γ-glutamylcysteine synthetase (glutamate cysteine ligase). The second step in GSH synthesis is regulated by glutathione synthetase. Glutathione cysteine ligase and cysteine (green) are the limiting factors in GSH synthesis. The γ-carboxyl linkage (gray) and the sulfhydryl group (green) provide stability and reductive power to the molecule, respectively. **(B)** Chemical structure of reduced (GSH), oxidized (GSSG) glutathione and GS-protein generated by protein glutathionylation. Glutathione peroxidase oxidizes GSH and glutathione reductase reduces GSSG, while glutathione-S-transferase participates in protein glutathionylation.

**FIGURE 2:**
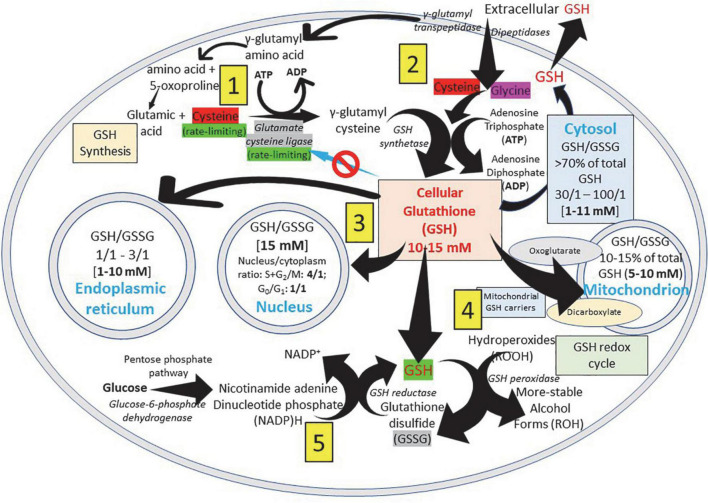
Glutathione distribution in subcellular compartments. GSH (γ- L-glutamyl-L-cysteinyl-glycine), a water-soluble tripeptide formed by the amino-acids glutamic acid, cysteine and glycine, is considered the major non-protein low molecular weight modulator of redox processes and the most important thiol reducing agent of the cell. (1) ATP-dependent GSH biosynthesis occurs in the cytosol of the cell and cysteine (red) and glutamate cysteine ligase (gray) are rate-limiting factors for its production. (2) Extracellular GSH is enzymatically degraded on the surface of the cells by γ-glutamyl transpeptidase generating the γ-glutamyl fraction (taken into the cell as γ-glutamyl-amino acid that can be metabolized to release the amino acid and 5-oxoproline, which can then be converted into glutamate to be used in the synthesis of GSH) and the cysteinyl-glycine fraction; and by dipeptidases splitting cysteinyl-glycine generating cysteine and glycine that are taken into the cell. (3) To allow normal cell function, it is essential to maintain an optimal GSH: GSSG ratio throughout all cell compartments. (4) The inner mitochondrial membrane system transport, that involves dicarboxylate and 2-oxoglutarate anion transporters, allows the passage of negatively charged GSH from the cytosol to the mitochondria. (5) GSH is present in both reduced (GSH) and oxidized (GSSG) states, and reduced GSH is maintained by GSH reductase, a cytosolic NADPH-dependent enzyme. GSSG returns to the reduced state by the NADPH-dependent activity of glutathione reductase. NADPH is rapidly regenerated from NADP + using electrons derived from catabolism of substrate molecules, such as glucose or isocitric and malic acid (pentose phosphate pathway). Reduced GSH neutralizes cellular hydroperoxides through GSH peroxidase activity.

The GSH (γ- L-glutamyl-L-cysteinyl-glycine) is a water-soluble tripeptide formed by the amino-acids glutamic acid, cysteine and glycine (Figure 1) present in the cytoplasm of all cells. GSH is found in all mammalian tissues as the most abundant non-protein thiol that defends against oxidative stress and possess a distinctive stability provided by a γ-carboxyl bond within the molecule (Figure 1A). GSH exists in the thiol-reduced and disulfide-oxidized (GSSG) forms (1, 2); and it’s free and bound to proteins (Figure 1). The reduced form GSH is the active form of the molecule, it is the most abundant and it is found inside the cells in millimolar concentrations in the range of 1–10 mM (highest concentration in liver) (5–8), while extracellularly they are found in micromolar (GSH in plasma: 10–30 μM) levels (5, 15, 16). The active group of the molecule is represented by the thiol group (-SH) of the cysteine residue (Figure 1) which provides its reductive power. The millimolar GSH intracellular concentrations, the low plasma micromolar concentrations and the low GSH redox potential (*E*′_0_ = −240 mV) make GSH an ideal and perfect cellular redox buffer (5, 16–18). GSH accounts for >98% of total GSH (3, 19–23). Eukaryotic cells have three major reservoirs of GSH. Most (80–85%) of the cellular GSH are in the cytosol, 10–15% is in the mitochondria and a small percentage is in the endoplasmic reticulum (Figure 2) (3, 24–28). GSSG is found mainly extracellularly. The redox state of the GSH/GSSG couple can serve as an important indicator of redox environment (29–31), and changes in this couple correlate with multiple cellular processes, including cell differentiation (32–37), cell proliferation (32–37), and cell apoptosis (38–42). GSH deficiency as evidenced by a decrease in the GSH/GSSG ratio manifests itself largely through an increased susceptibility to oxidative stress, and the resulting damage is thought to be involved in SARS-CoV-2 infection leading to COVID-19 disease (6, 7, 39, 43–53). In addition, imbalances in GSH levels affect immune system function, and are thought to play a role in the aging process and the diseases of aging, one of the principal risk factors for the development and progression of COVID-19 disease.

**FIGURE 3:**
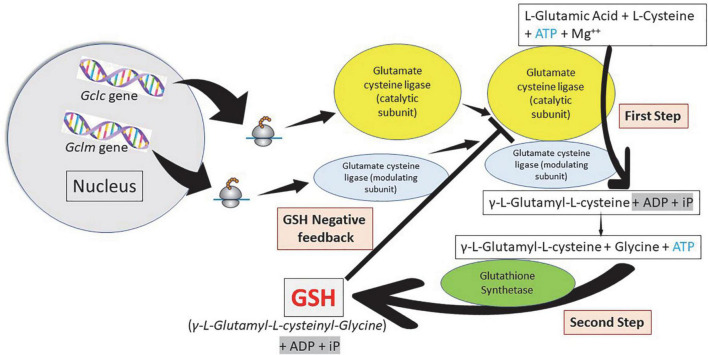
Glutathione synthesis: A two-step pathway. Homeostasis of cellular glutathione. Synthesis and regulation of the cell concentrations. Glutamate cysteine ligase (γ-glutamyl cysteine synthetase) constitute the first step in the synthesis of glutathione (GSH) forming γ-L-glutamyl-L-cysteine using adenosine triphosphate (ATP). Glutathione synthetase constitute the second step forming GSH, also using ATP. Cellular GSH concentration regulates the function of glutamate cysteine ligase.

**FIGURE 4:**
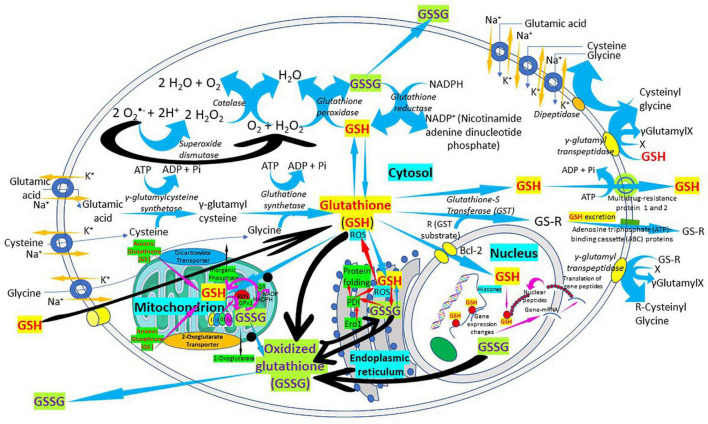
The “Glutathione Pathway.” *Glutathione synthesis*, γ*-glutamyl pathway, cellular distribution, antioxidant properties, catabolism of xenobiotics*, and *glutathione recycling in the cell*. The figure shows a schematic representation of the “glutathione pathway.” Glutathione (GSH) is synthesized from glutamate, cysteine, and glycine by γ-glutamyl-cysteine synthetase (glutamate cysteine ligase) and glutathione synthetase. Glutathione redox state is regulated, in part, by glutathione peroxidases, forming oxidized glutathione (GSSG), and by a reaction catalyzed by glutathione reductase. Glutathione is conjugated to substrates both through the action of the glutathione S-transferases and through non-enzymatic reactions. Glutathione conjugates can be excreted from the cells by members of the ATP-binding cassette (ABC) transporter family.

Glutathione is synthesized in the cytosol of all cells from their precursor amino acids: glutamic acid, cysteine and glycine by consecutive action of two enzymes: γ-glutamyl-cysteine (γ-GluCys) synthetase (also known as glutamate cysteine ligase, GCL) that in a first step uses glutamate and cysteine as a substrate to form the dipeptide γ-glutamyl-cysteine; and glutathione synthetase that in a second step combines γ-glutamyl-cysteine with glycine for forming GSH (54, 55) (Figure 3). ATP (adenosine triphosphate) acts as a co-substrate for both enzymes (Figures 2, 3). Under normal physiological conditions, the rate of synthesis of GSH is determined to a large extent by two factors: (a) the *activity of GCL* and (b) the *availability of the cysteine substrate*. Therefore, the intracellular levels of GSH are regulated by the negative feedback of GSH itself on the GCL enzyme (1, 4, 55–57) and by the availability of the amino acid L-cysteine (1, 4, 58). The GCL enzyme is a heterodimer formed by two subunits: the heavy subunit or glutamate cysteine ligase catalytic subunit (GCLC, 73 kDa) and the light subunit or glutamate cysteine ligase modulating subunit (GCLM, 30 kDa). The heavy subunit has the active site responsible for the union between the amino group of the cysteine and the γ-carboxyl group of glutamate. The GCLM subunit has no enzymatic activity but has an important regulatory function increasing the efficiency of the GCLC subunit. This subunit is required for optimal activity and feedback inhibition by GSH (59). GSH inhibits GCL by competing with glutamate in the active site of GCLC (1, 57–60). The enzyme glutathione synthetase (GS) is formed by two identical subunits (52 kDa/subunit) and is not regulated by intracellular levels of GSH. The active site of the enzyme that binds glycine to the dipeptide γ-L-glutamyl-L-cysteine is highly specific (57). GCL is considered the speed limiting enzyme of synthesis since overexpression of GS does not increase GSH levels while overexpression of GCL increases the synthesis of GSH (61) (Figure 3). ATP is the energy donor for both enzymes. As mentioned above, GSH cellular concentrations are regulated by GSH-mediated GCL inhibition (Figures 2, 3). Thus, the biological control of intracellular GSH homeostasis through consumption and supply is an intricately balanced process that prevents oxidative stress. Cellular GSH (cytosol, mitochondria, endoplasmic reticulum, nucleus; Figures 2, 4) availability is maintained by *de novo* synthesis from precursor amino acids, (glutamate, cysteine, and glycine), reduction of GSSG by glutathione reductase (GR), and uptake from exogenous GSH sources across plasma membranes (Figure 4) (62, 63). The three amino acids are adsorbed by transporters. Additionally, intestinal epithelial cells can import intact GSH from the lumen *via* specific plasma membrane transporters (7).

### Glutathione cellular distribution

Glutathione is found in almost all cellular compartments, including the nucleus (5, 54, 64–68) (Figures 2, 4). The GSH transport between the various cell compartments is vital to buffer reactive oxygen species (ROS) and facilitate redox signaling in order to control cell growth, development and defense, as well as regulate cell proliferation. GSH is predominantly in its thiol-reduced form inside the cells, except in the lumen of the endoplasmic reticulum where it exists only in its GSSG form (Figures 2, 4). The GSH content existing in millimolar concentrations varies among different organs; liver being among organs with the highest content (56). GSH content also varies among different areas of the same tissues; periportal hepatocytes may contain nearly twice the centrilobular concentration, enterocytes at the villus tip have a higher content than the crypts, and renal proximal tubular cells have more GSH than other parts of the nephron (56). Mitochondria contain 10–15% of the intracellular GSH reaching a concentration of 10–12 mM (54) while in the cytosol the concentration is 7 mM (54, 56). This difference in concentration is associated with the absence of catalase inside the mitochondria, what leaves GSH in charge of all inactivation of the hydrogen peroxide generated during the oxidative processes that occur in the mitochondrial matrix (57).

The concentration of GSH in the mitochondrial compartment is more important for cell survival than the GSH found in the cytosol. Since mitochondria do not have the enzymes involved in the synthesis of GSH, all the GSH found in the mitochondrial compartment comes from the cytosol. A system transport present in the inner mitochondrial membrane, that involves dicarboxylate and 2-oxoglutarate anion transporters, allows the passage of negatively charged GSH from the cytosol to the mitochondria. The first incorporates GSH into the mitochondria by inorganic phosphate exchange and the second by exchange of 2-oxoglutarate (27, 28, 64) (Figures 2, 4). While the greater amount of cellular reduced GSH is found in the cytosol and mitochondria, the endoplasmic reticulum becomes a reservoir of small concentrations of the oxidized form of GSH (GSSG). The ratio of reduced GSH to the disulfide form (GSH/GSSG) within the endoplasmic reticulum ranges from 1:1 to 4:1, whereas the overall cellular GSH/GSSG ratio ranges from 30:1 to 100:1 (26) (Figure 2). There is a preferential transport of GSSG from the cytosol to the endoplasmic reticulum to maintain an adequate environment for protein disulfide bond formation and protein folding (69–71). There is little data about the concentrations of GSH in the nucleus and endoplasmic reticulum largely because of a lack of adequate techniques to accurately determine the GSH pool at those locations (15, 69, 72, 73). There are great variations in nuclear GSH concentration and its regulation mechanisms during the cell cycle since cells starting the proliferation phase have high levels of nuclear GSH, while resting cells have similar or lower GSH levels in the nucleus than in the cytoplasm (68, 72, 73). High nuclear GSH concentrations are vital since increase in total GSH is necessary for the cells to progress from the G1- (with low GSH levels) to the S-phase; addition of GSSG causes the cell cycle to arrest at G1; and excessive and prolonged oxidation arrest cell cycle triggering cell death (68, 72, 73). GSH behaves as “redox sensor” at the DNA synthesis onset by maintaining nuclear architecture providing the appropriate redox environment for DNA replication and safeguarding DNA integrity (72) and is a key regulator of epigenetic events critical in cell proliferation regulation and cellular resistance to apoptosis (73).

### Glutathione and the γ-glutamyl cycle

**FIGURE 5:**
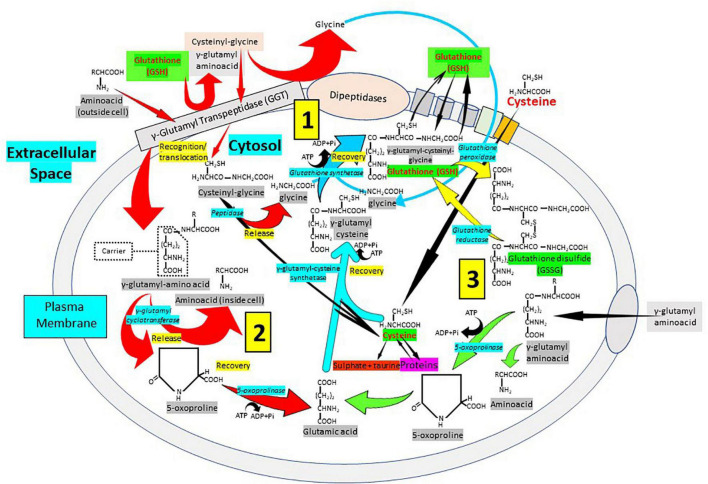
Cellular glutathione synthesis and recycling: The importance of the γ-glutamyl pathway. The degradation or catabolic part of the GSH cycle, takes place partially extracellularly and partially inside cells. (1) The extracellular degradation of GSH occurs on the surface of the cells that express the enzyme γ-glutamyl transpeptidase (GGT) and the dipeptidases found in the external plasma membrane. Following plasma membrane carrier-mediated GSH release from the cell, GSH becomes accessible to the active site of γ*-*glutamyl transpeptidase, which catalyzes GSH breakdown into γ-glutamyl fraction and cysteinyl-glycine by transferring the γ-glutamyl fraction to an amino acid acceptor, forming γ-glutamyl-amino acid. The cysteinyl-glycine fraction is split by the enzyme dipeptidase generating cysteine and glycine. (2) The γ-glutamyl-amino acid can be metabolized to release the amino acid and 5-oxoproline, which can then be converted into glutamate to be used in the synthesis of GSH. (3) The cells incorporate cysteine and most of the intracellular cysteine is used for the synthesis of GSH. Cysteine can be used for protein synthesis and part can be degraded to sulfate and taurine. The cycle γ-glutamyl allows GSH to be used as a continuous source of cysteine. The γ-glutamyl amino acid is taken up by cells through a specific transport mechanism. Cysteinyl glycine is also taken up by cells. Inside the cell, the γ-glutamyl amino acid is hydrolyzed by γ-glutamyl cyclo-transferase and converted into oxoproline, a cyclic form of glutamate converted into glutamate *via* oxoprolinase, and a free amino acid.

The synthesis, transport and catabolism of GSH occur in a series of enzymatic steps and transports of membrane that are collectively called γ*-glutamyl cycle* (Figure 5) (1, 74, 75). The γ*-*glutamyl cycle was postulated by Meister (76) and it accounts for the GSH biosynthesis and degradation. The GSH biosynthesis has been described previously. After its synthesis, GSH is transported to the intracellular compartments, mitochondria, endoplasmic reticulum and nucleus, but most of it is released through transporters toward the extracellular space. In contrast to the synthesis, that occurs only intracellularly, the degradation or catabolic part of the GSH cycle, takes place partially extracellularly and partially inside cells. The extracellular degradation of GSH occurs on the surface of the cells that express the enzyme γ-glutamyl transpeptidase and the dipeptidases found in the external plasma membrane (1) (Figure 5). After the plasma membrane carrier-mediated GSH release from the cell, GSH becomes accessible to the active site of γ*-*glutamyl transpeptidase, which catalyzes the breakdown of the GSH γ*-*glutamyl bond forming two fractions: The γ-glutamyl fraction and the cysteinyl-glycine by transferring the γ-glutamyl fraction to an amino acid acceptor, forming γ-glutamyl-amino acid. Once inside the cell, the γ-glutamyl-amino acid can be metabolized to release the amino acid and 5-oxoproline, which can then be converted into glutamate to be used in the synthesis of GSH. On the other hand, also in the extracellular space, the cysteinyl-glycine fraction is split by the enzyme dipeptidase generating cysteine and glycine. The cells incorporate cysteine and most of the intracellular cysteine is incorporated into the synthesis of GSH. Depending on the metabolic needs of the cell, the cysteine can be used for protein synthesis and part can be degraded to sulfate and taurine. The cycle γ-glutamyl allows GSH to be used as a continuous source of cysteine. The γ-glutamyl amino acid is taken up by cells through a specific transport mechanism. Cysteinyl glycine is also taken up by cells. Inside the cell, the γ-glutamyl amino acid is hydrolyzed by γ-glutamyl cyclo-transferase and converted into oxoproline and a free amino acid. Oxoproline is a cyclic form of glutamate and is converted into glutamate *via* oxoprolinase (Figure 5). The γ-glutamyl cycle was initially postulated by Meister as a mechanism for amino acid transport (76). However, this presents major problems. The most important is the energetic one. The γ-glutamyl cycle requires the use of three ATP molecules per turn of the cycle. Thus, the uptake of an amino acid would require the use of three high-energy phosphate bonds. In favor of the cycle was the fact that addition of γ-glutamyl transpeptidase inhibitors *in vivo* caused a decrease in amino acid transfer into cells. The gamma-glutamyl cycle should be considered not as a mechanism for amino acid transport but rather a generator of extracellular signals, gamma-glutamyl amino acids, that are converted intracellularly to 5-oxoproline, which activates uptake and/or metabolism of amino acids (1, 74, 75). The γ*-*glutamyl amino acids or oxoproline could be signaling molecules to activate the transport of amino acids through membranes. Oxoproline catalytically activates the uptake of amino acids through the placental barrier, and the transfer of amino acids through the blood–brain barrier is activated by oxoproline (2, 75). Thus, the γ-glutamyl cycle, apart from explaining the synthesis and degradation of glutathione, may serve as a generator of signals to activate amino acid transport into cells (2, 75). GSH turnover may be considered as a multi-organ process. In fact, in liver, an organ in which glutathione synthesis is most active, the degradation is very slow due to the very low activity of γ-glutamyl transpeptidase. In the kidney, however, γ-glutamyl transpeptidase is very high. Thus, the γ-glutamyl cycle may be considered as a multi-organ cycle in which the synthetic part occurs in liver and the catabolic part occurs in kidney amongst other tissues.

### Glutathione and damaging inflammation in lower respiratory diseases

**FIGURE 6:**
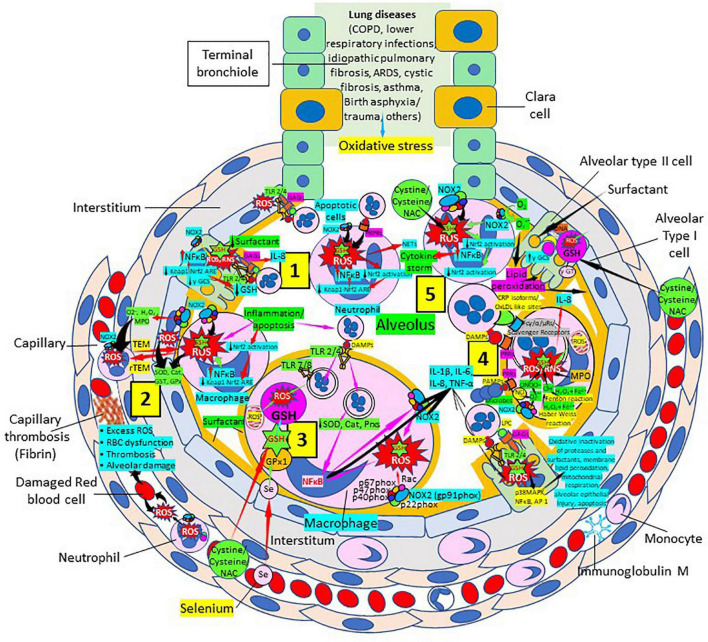
Oxidative stress, reduced glutathione (GSH) and lung diseases. (1) Lung diseases affect alveolar cells increasing reactive oxygen species (ROS) production, reduce Kelch-like ECH-associated protein 1 (Keap1)-Nuclear factor erythroid 2-related factor 2 (Nrf2)-antioxidant response element (ARE) redox regulator pathway and become defective for surfactant production. Damaged/apoptotic cells cause alveolar cell activation of nuclear factor (NF)-κB and release cytokines like interleukin (IL)-8. Alveolar type I cells augment ROS production *via* toll-like receptors (TLRs) 1 and 2. Inflammation enhances neutrophil extracellular trap (NET) release and increases ROS production. (2) Inflammation associated to lung diseases augments macrophage’s ROS production, inhibiting Nrf2 activation and enhancing NF-κB upregulation. ROS are counterbalanced by enzymes like superoxide dismutase (SOD), catalase (Cat), glutathione S-transferase (GST), and glutathione peroxidase (GPx) to protect cells from oxidative damage caused by nicotinamide adenine-dinucleotide phosphate (NADPH) oxidase 2 (NOX2), superoxide (O_2_^–^), hydrogen peroxide (H_2_O_2_), and myeloperoxidase (MPO). Capillary neutrophils migrate to and from alveoli by *trans-*endothelial (TEM) and reverse transmigration (rTEM), respectively. Inflammation can cause excessive ROS production in capillaries, red blood cell (RBC) dysfunction, thrombosis and alveolar damage. (3) Activated alveolar macrophages release increased levels of IL-1β, IL-6, IL-8, and tumor necrosis factor (TNF)-α. Inflammation-associated activated macrophages (*via* TLRs) reduce enzymes like SOD and Cat, among others, and activate NF-κB. NOX2 activation increases ROS production that enhance NF-κB activation. Glutathione (GSH) precursors (Cystine, cysteine, N-acetyl cysteine, NAC), and selenium (Se) restore GSH and GPx, respectively, to counteract the effects of ROS. (4) Alveolar macrophages engulf microbes and apoptotic cells *via* Fc (γ/α/μ) and scavenger receptors and/or pattern recognition protein receptors (PRPRs) leading to increased ROS production and cytokine release. MPO, nitric oxide (NO), O_2_^–^, and H_2_O_2_ through the Fenton and Haber-Weiss reactions that generate hydroxyl radicals, participate in ROS and RNS generation. Lung disease-associated inflammation and apoptosis [*via* TLRs and glycosaminoglycans (GAGs)] enhance alveolar cell ROS production that *via* p38MAPK, NF- κB, and AP-1 activation, contribute to epithelial injury and further inflammation. (5) Neutrophils contribute to O_2_^–^ production, lipid peroxidation and increased oxidative stress to promote a cytokine storm (249). Administration of GSH precursors [cystine, cysteine, NAC; see (3), (4), and (5)] facilitate GSH formation to reduce oxidative stress. Abbreviations: PRRs, pattern recognition receptors; ɣ-GCS, ɣ-glutamyl cysteine synthetase; DAMPs, damage associated molecular patterns; Prxs, peroxiredoxins; NAC, N-acetyl cysteine; ɣ-GT, ɣ-glutamyl transpeptidase; PAMPs, pathogen associated molecular patterns; LPC, lysophosphatidylcholine.

Glutathione Samsonian (mighty) power is centered in the thiol (sulfhydryl) group of the cysteine amino acid. GSH participates in numerous key processes where the thiol reducing potential is utilized. Several lung disorders are believed to be characterized by an increase in alveolar oxidant burden, potentially depleting alveolar and lung GSH. Low GSH has been linked to abnormalities in the lung surfactant system and the interaction between GSH and antiproteases in the epithelial lining fluid of patients. Normal levels of intracellular GSH may exert a critical negative control on the elaboration of proinflammatory cytokines. The increase of intracellular ROS is believed to correlate with the activation of nuclear factor (NF)-kappa B, a transcription activator linked to the elaboration of several cytokines (Figure 6). There is now sufficient data to strongly implicate free radical injury in the genesis and maintenance of several lung disorders in humans. This information is substantial and will help the development of clinical studies examining a variety of inflammatory lung disorders.

Oxidative stress and inflammation are considered fundamental mediators of chronic obstructive pulmonary disease (COPD) pathophysiology (77–91). The lungs are directly exposed to tobacco smoke and air pollutants that are main sources of ROS. ROS directly cause lung damage as a result of DNA, lipid, carbohydrate, and protein alterations, and activate local inflammatory responses that contribute to COPD development and progression (79–83). ROS can further activate epithelial cells and macrophages facilitating neutrophil, monocyte, and lymphocyte recruitment, and the recruited activated inflammatory cells subsequently enhance additional ROS generation, increasing the pro-oxidant burden (80–85). ROS and RNS production are facilitated by pattern recognition receptors [C-reactive protein (CRP), toll-like receptors] capable of recognizing pathogen (bacterial/viral)-associated molecular patterns and/or damage-associated molecular patterns in apoptotic or damaged cells (92–100) (Figure 6). The phosphocholine head group in phospholipids of normal healthy cell membranes is not accessible but, when cells are damaged and die, enhanced availability of lysophosphatidylcholine and disruption of the lipid bilayer expose phosphocholine residues to which CRP avidly binds (99). These events lead to a state of persistent inflammation and chronic oxidative stress (82–85), characterized by increased ROS production, reduced GSH peroxidase activity, selenium deficiency and reduced GSH levels (80–91). Asymptomatic smokers and stable COPD patients showed increased GSH levels in bronchoalveolar lavage, while patients with severe/very severe exacerbation periods had significantly decreased levels (101). Patients with decreased GSH and increased oxidative stress also showed increased neutrophil influx and IL-8 levels (101). Alveolar macrophages derived from circulating monocytes recruited into the lungs by monocyte chemotactic factors produced by lung cells are increased 20-fold in COPD patients and release ROS as superoxide anions and hydrogen peroxide (102). Antioxidant therapies should be effective in preventing COPD disease progression and exacerbations. Although prolonged treatment with oral N-acetylcysteine (NAC) prevents acute exacerbations of chronic bronchitis, it remains controversial for the treatment of COPD (91, 103–105). A combination of antioxidants including thiol-based antioxidants, mitochondria-targeted antioxidants and Nrf2 activators should be more effective in the treatment of COPD patients (91).

In acute respiratory distress syndrome (ARDS), there is extensive overproduction of free radicals and reduced extracellular and intracellular GSH leading to oxidative cell damage (106). ROS such as hydrogen peroxide and hypochlorous acid may play a key role in the pathogenesis of the acute lung injury (107). It has been shown that alveolar epithelial lining fluid of patients with ARDS is deficient in total GSH compared to normal subjects (107), and neutrophil-mediated oxidants release leads to GSH deficiency and lung cell injury (107). The global antioxidant capacity of the epithelial lining fluid, despite an increase in single antioxidant compounds, seems unable to fully counterbalance the increased oxidative burden (108). NAC benefited ARDS patients as evidenced by intracellular (inside red blood cells) and extracellular (plasma) antioxidant defense biomarkers and outcome. NAC treatment increased extracellular total antioxidant power and total thiol molecules and enhanced intracellular GSH and patients’ outcome (106). NAC treatment improved oxygenation and decreased mortality in ARDS patients; and patients with glutathione-S-transferase M1 (proinflammatory-cytokine producer macrophages) null and M1 and T1 (Type 1 helper CD4 + lymphocytes) double null polymorphisms had increased mortality suggesting that antioxidant therapy becomes fundamental for those patients (109). A depressed antioxidant defense and dysfunctional iron regulation in ARDS might cause greater inflammation and anemia (110).

Glutathione is an important antioxidant in the lungs, but its concentration is low in the airways of patients with cystic fibrosis, since GSH is transported into the airways by the cystic fibrosis transmembrane conductance regulator, which is mutated in cystic fibrosis patients (111). The concentration of GSH that is normally about 400 μM in the epithelial lining fluid, over a 100-fold higher than in plasma, is low in the airways of patients with cystic fibrosis from an early age (112–114). Extracellular glutathione S-transferase omega-1, a cytosolic enzyme that modulates the S-thiolation status of intracellular factors involved in the inflammatory response, and its polymorphisms have been associated with an increased risk to develop COPD and could have a biological and clinical significance in cystic fibrosis (115). Low GSH, neutrophil infiltration, myeloperoxidase activity and inflammation increase oxidative stress overwhelming the antioxidant defense, and hypochlorous acid mediated GSH oxidation and its attachment to proteins contribute to further GSH deficiency (114). The lack of efficacy of inhaled GSH in patients with cystic fibrosis could be explained by the high concentrations of the GSH-degrading enzyme γ-glutamyltransferase present in lung fluids of those patients (116–123), and then, the use of precursors of GSH synthesis like NAC and cystine could be more effective in the synthesis of GSH (124). Lack of oral GSH supplementation effects upon growth or changes in serum or fecal inflammatory markers in children with cystic fibrosis with pancreatic insufficiency (125) could be probably explained by the inability of the cells to uptake extracellular GSH to be used inside the cells. Decreased GSH content in the apical fluid in cystic fibrosis could be the result of abnormal GSH transport associated with a defective cystic fibrosis transmembrane conductance regulator as mentioned previously (126).

An oxidant/antioxidant imbalance characterized by oxidative stress and low GSH levels is involved in the pathogenesis of idiopathic pulmonary fibrosis, since data show marked GSH deficiency in the lower respiratory tract of those patients (127). Glutathione-S-transferase π (GSTP) that participates in the conjugation of GSH to reactive cysteines (S-glutathionylation) seems to play an important role in idiopathic pulmonary fibrosis lung fibrogenesis, since GSTP immunoreactivity is increased in the lungs of idiopathic pulmonary fibrosis patients, notably within type II epithelial cells (128, 129). GSTP inhibition *via* the airways may be a novel therapeutic strategy for the treatment of idiopathic pulmonary fibrosis (128, 129). The use of GSH precursors like N-acetyl cysteine, enhancers of nuclear factor erythroid 2-related factor 2 (Nrf2) like sulforaphane, melatonin, and many more molecules involved in antioxidant defense were proposed as supplementation of other idiopathic pulmonary fibrosis therapies (130). Inhaled (nebulized or aerosolized) reduced GSH to augment the deficient GSH levels of the lower respiratory tract has been used effectively in numerous pulmonary diseases and respiratory conditions like HIV seropositive individuals, cystic fibrosis and idiopathic pulmonary fibrosis, among others (131–133). GSH has clearly a regulatory role in inflammation and immunity (134). GSH acts as an inhibitor of extended inflammation directing components of innate immunity like polymorphonuclear neutrophils specifically to the site of infection/damage allowing a proper response to infection. GSH then directs the migration of inflammatory polymorphonuclear neutrophils away from the lung, where they cause ARDS, and toward the site of infection, where they kill microorganisms. As a result, it develops more immunity and less inflammation, with the concomitant increased survival; in addition, GSH becomes not just an inhibitor of inflammation but a regulator of innate immunity in a direction that benefits the host (134).

### Glutathione and atherosclerosis

Cardiovascular diseases are the leading causes of death in the US compared to any other cause (135). Cardiovascular complications are thought to result from increased free radical levels that impair redox homeostasis, that represents the interaction between oxidative stress and reductive stress. A prolonged oxidative or reductive stress will alter the homeostatic redox mechanism to cause cardiovascular complications. GSH, the most abundant antioxidant in the heart, plays a fundamental role in normalizing a redox homeostatic mechanism that was shifted toward oxidative or reductive stress. This may lead to impairment of cellular signaling mechanisms and accumulation of misfolded proteins causing proteotoxicity associated with cardiac dysfunction (136–143). Oxidative stress is crucial in atherogenesis (144–151), suggesting that a specific antioxidant/prooxidant imbalance, characterized by a weak GSH-related enzymatic antioxidant shield present in human atherosclerotic lesions, may be involved in atherogenic processes in humans (152). A higher level of oxidative stress as evidenced by elevated plasma malondialdehyde levels and low levels of GSH, α-tocotrienol and GSH peroxidase activity in patients under 45 years old may play a role in the development of premature coronary artery disease and be potential biomarkers for premature coronary artery disease (153). Similarly, coronary artery disease patients with single, double, or triple-vessel stenosis and patients with acute coronary syndrome had a significant increase in malondialdehyde levels and the percentage of malondialdehyde release, associated with a marked decrease in GSH concentration, total antioxidant capacity and erythrocyte GSH peroxidase activity compared with controls (154). Interestingly, differences in prooxidative parameters were more profound in acute coronary syndrome patients compared with coronary artery disease patients indicating that the acute form of coronary artery disease is more susceptible to oxidative damage, suggesting that use of antioxidant therapy may be warranted to reduce oxidative stress in this disorder (154).

Glutathione might inhibit the effects of cerebral infarction and enhance antiapoptotic signaling after ischemic stroke, suggesting that GSH may be a potent therapeutic antioxidant that can attenuate severe pathologies after ischemic stroke, and stimulating GSH synthesis through administration of GSH precursors and micronutrients like selenium can optimize GSH and GSH peroxidase for optimal antioxidant defense in cerebral ischemia (155, 156). Low total GSH and high homocysteine levels are considered as novel risk markers for acute stroke severity, and low total and reduced GSH levels may be potential risk markers for stroke severity and insufficient functional independence in large-artery atherosclerosis (157, 158). Since GSH is the final product of the homocysteine metabolism in the transsulfuration pathway by transferring sulfur from homocysteine to cysteine, a deficiency in transsulfuration pathway leads to excessive homocysteine production (hyperhomocysteinemia) and reduced GSH synthesis (159, 160). Homocysteine is a sulfur-containing amino acid tightly involved in methionine metabolism. Indeed, if there is a methionine deficit, homocysteine can be re-methylated to form methionine, and if there is an adequate amount of methionine, homocysteine is used to produce cysteine (161). Hyperhomocysteinemia decreases GSH peroxidase activity leading to the prevalence of GSSG on GSH with the GSH/GSSG impaired ratio causing some common cardiovascular and neurodegenerative disorders (159). N-acetyl-cysteine administration supplies the cysteine necessary for GSH synthesis and concomitantly reduces hyperhomocysteinemia, improving GSH peroxidase activity and reducing oxidative stress (159). Furthermore, the well documented efficacy of combined folic acid, B6, and B12-vitamin supplementation to reduce hyperhomocysteinemia could enhance GSH activity and reduce oxidative stress (161). Recently, it was shown that cysteine uptake *via* excitatory amino acid carrier 1 suppresses ischemia-induced neuronal death through promotion of hippocampal GSH synthesis in ischemic animal models (162). Alterations in the normal function of excitatory amino acid carrier 1 affect cysteine transport and GSH synthesis impairing zinc homeostasis (the thiol group of GSH can function as a principal Zn^2+^ chelator for the maintenance of Zn^2+^ homeostasis in neurons) and oxidative stress, enhancing susceptibility to ischemia-induced neuronal cell death in the hippocampus (162–164). Increased GSH synthesis neutralizes reactive oxygen and nitrogen species and regulates zinc homeostasis promoting neuroprotection after ischemia/reperfusion (162).

**FIGURE 7:**
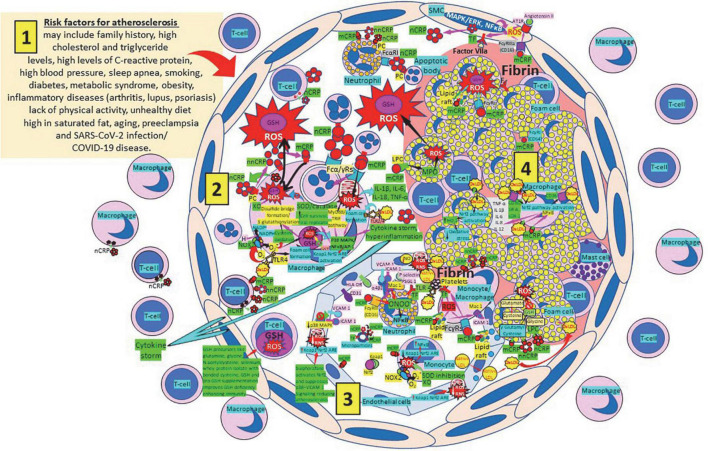
Oxidative stress, reduced glutathione (GSH) and atherosclerosis. (1) Risk factors for atherosclerosis. (2) Atherosclerosis risk factors facilitate oxidative stress and inflammation in the arterial intima. Native C-reactive protein (nCRP), a pattern recognition receptor produced in the liver, macrophages, lymphocytes, smooth muscle cells (SMC), and other cells, promotes inflammation through monomeric CRP (mCRP) enhancing intimal oxidative stress. Oxidized (ox) LDL binds macrophage toll-like receptor (TLR) 4 and facilitates nicotinamide adenine dinucleotide phosphate (NADP)H oxidase 2 (Nox2) activity and superoxide (O_2_^–^) production causing cysteine oxidation, disulfide bridge formation and S-glutathionylation. Xanthine oxidase (XO) and inhibition of superoxide dismutase (SOD)/catalase further facilitate O_2_^–^ cellular activity. OxLDL bound to TLRs 2 and 4 promotes foam cell formation and activates transcription factors like nuclear factor (NF)-κB facilitating cytokine storm and hyperinflammation. Excessive mitochondrial reactive oxygen species (ROS) generation further enhances cytokine production. CRP (nCRP, mCRP) can facilitate macrophage and neutrophil uptake of apoptotic cells through Fcγ and Fcα receptors, respectively (FcRs). Oxidative stress also activates the Kelch-like ECH-associated protein 1 (Keap1)-Nuclear factor erythroid 2-related factor 2 (Nrf2)-antioxidant response element (ARE) redox regulator pathway in monocytes [see (3) and macrophages (2)], releasing Nrf2 to regulate the expression of genes that control antioxidant enzymes like glutathione S-transferase (GST), facilitating glutathione (GSH) activity. Macrophages, T-lymphocytes, neutrophils and SMCs can generate mCRP increasing inflammation. (3) Monocytes, macrophages, neutrophils, endothelial cells and microparticles can generate mCRP, increase O_2_^–^ and ROS formation and reactive nitrogen species like peroxinitrite (ONOO^–^), and tissue factor (TF) expression enhancing oxidation, inflammation and thrombosis. TLR 4-mediated oxLDL-binding to platelets promotes thrombosis; mCRP binding to lipid rafts and FcγRs enhances inflammation; and endothelial activation allows intimal cell migration. GSH enhancement and Nrf2 activation augment immunity and reduce atherosclerosis. (4) Foam cells and smooth muscle cells associated with atherosclerotic plaques enhance ROS formation, cytokine release and tissue factor (TF)-mediated fibrin deposition. Abbreviations: MAPK/ERK, mitogen-activated protein kinases/extracellular signal-regulated kinases; AT1R, angiotensin II type 1 receptor; PC, phosphorylcholine; LPC, lysophosphatidylcholine; MPO, myeloperoxidase; nnCRP, non-native CRP; TNF, tumor necrosis factor; IL, interleukin; ACE, angiotensin converting enzyme; MyD88/TRIF, myeloid differentiation primary response 88/TIR-domain-containing adapter-inducing interferon-β; PI3K/Akt, phosphatidylinositol-3-kinase/protein kinase B; MAPK, mitogen-activated protein kinase; AP-1, activator protein 1; CD31, cluster of differentiation 31; ICAM-1, intercellular adhesion molecule-1; Mac-1, macrophage-1 antigen; PSGL-1, P-selectin glycoprotein ligand-1; HLA-DR, human leukocyte antigen–DR isotype.

Atherosclerosis represents a state of intense oxidative stress characterized by vascular wall lipid and protein oxidation that contributes to chronic inflammation within the arterial wall, in which CRP is a major player (Figure 7). The balance of the different CRP isoforms, monomeric (mCRP) or native pentameric (nCRP) within the plaque determines the preponderance of a proinflammatory or anti-inflammatory effect, respectively (165). CRP is synthesized in smooth muscle cells of atherosclerotic lesions with active disease, foam cells, macrophages, lymphocytes, monocytes, and endothelial cells within the atherosclerotic plaque (166–170). CRP binds and aggregates oxidized low-density lipoprotein (ox-LDL) and enhances macrophage oxLDL uptake, promoting mitogen-activated protein kinase activation (171) required for foam cell formation (172). OxLDL enhances toll like receptor 4 expression further facilitating foam cell formation and development and progression of atherosclerosis (173, 174). CRP binding to oxLDL and apoptotic cells occurs through phosphorylcholine, and binding to this ligand starts phagocytosis (100, 170, 175–181). The different CRP isoforms, nCRP, non-native pentameric CRP (nnCRP) and mCRP (175–178, 181–184), may explain their protective and destructive effects, with nCRP being primarily antiinflammatory inducing Th2/M2 responses, while mCRP being typically proinflammatory inducing Th1/M1 responses (170, 185–189). Pentameric nCRP and CRP peptides 77–82, 174–185, and 201–206 can control the inflammatory response resolving inflammation by reducing inflammatory cell endothelial adhesion and tissue migration, and the described CRP-mediated enhanced monocyte chemotaxis could be explained by local generation of mCRP (190, 191). Pro-inflammatory and proatherogenic mCRP, but not nCRP, induces ROS monocyte/macrophage production and facilitates macrophage uptake of necrotic cells (170, 192) contributing to foam cell formation, atherosclerotic plaque formation and plaque rupture or destabilization (190, 191). Foam cell formation during atherogenesis could be also explained in part by uptake of CRP-opsonized native LDL (193). The dissociation/relaxation of nCRP into nnCRP occurs on necrotic, apoptotic, and ischemic cells, membranes of activated platelets, monocytes, and endothelial cells, and on the surface of microparticles, *via* phosphorylcholine binding and seems to be, as mCRP, proinflammatory (194–196). Pentameric nCRP does not possess intrinsic proinflammatory properties, while nnCRP and mCRP do (170, 196). The mCRP isoform, unlike nCRP, has a stimulatory effect on platelets, facilitates thrombus growth through platelet stimulation, and is the more potent reagent, both increasing monocyte activation and ROS production, generated through myeloperoxidase-mediated respiratory burst and raft-associated reduced nicotinamide adenine dinucleotide phosphate (NADPH)-oxidase during oxLDL-mediated foam cell formation (100, 170, 197–204). ROS activity in the vessel wall contributes to the formation of oxidized LDL, a major contributor to the pathogenesis of atherosclerosis (205, 206). Thrombus formation and the subsequent activation of the coagulation cascade with final generation of fibrin is facilitated by the mCRP-mediated enhancement of tissue factor on the endothelial cell surface, platelet aggregation and thrombus growth (207, 208) (Figure 7). OxLDL components and their interaction with toll-like receptors (TLRs) 2 and 4, CD36 and other cellular receptors further mediate thromboinflammation enhancing tissue and organ damage culminating in organ failure, i.e., myocardial infarction, stroke, and pulmonary artery embolism (174, 209–212).

The strong role of severe oxidative stress, reduced antioxidant defenses like GSH with increased lipid peroxidation and malondialdehyde generation (213), lipid, protein and DNA oxidation with increased apoptosis and necrosis in atherosclerosis as a major cause of cardiovascular diseases and stroke, supports the use of complementary and alternative medicines, dietary supplements, and antioxidants with hardly any adverse effect, able to restore homeostasis reversing oxidative stress (214). Enhancing GSH synthesis, selenium levels and redox-active selenoproteins, and activating Nrf2 and other antioxidant enzymes will strengthen the cardiovascular antioxidant defense. Phenolic compounds like phenolic acids, flavonoids, lignans and tannins can limit LDL oxidation and foam cell formation (215). Selenium is an essential micronutrient that modulates cardiovascular functions *via* its incorporation into selenoproteins as the amino acid selenocysteine (216, 217). Intravenous reduced GSH supplementation reverses endothelial dysfunction in patients with atherosclerosis enhancing NO activity and NO-mediated vasodilation (218). GSH stores and transports cysteine, and cysteine forms less diffusion-limited NO adducts that may transport NO to reach sites within vascular smooth muscle cells and platelets (218, 219). Since GSH is not carried inside the cell, exogenously administered GSH is most likely to act by increasing plasma GSH levels reducing luminal oxidative stress and increasing NO bioavailability in patients with endothelial dysfunction (220).

Administration of GSH precursors like cysteine/N-acetylcysteine, glycine and/or glutamic acid will facilitate the synthesis of GSH within each cell of the body including the atherosclerotic plaque, reducing ROS and LDL oxidation, enhancing NO production, and mitigating atherosclerosis and its complications (221–229). Considering the paramount importance of oxLDL in the pathogenesis of atherosclerosis, it is reasonable to evaluate the role of antioxidants in the treatment of the disease as adjuvant strategies to lipid-lowering or anti-inflammatory therapies designed to reduce the risk of cardiovascular disease (230). Since oxidation participates as an essential messenger of cellular signaling pathways, treatment of oxidative stress needs to consider maintaining that physiologic threshold (230, 231). The lack of standardized methods to evaluate total antioxidant capacity and the oxidation state and the use of inadequate antioxidants and/or improper concentrations of antioxidants lead to failure of numerous clinical trials directed to prevent or mitigate progression of atherosclerosis (230–233).

Nuclear factor erythroid 2-related factor 2 plays a fundamental role in the response to oxidative stress and xenobiotic metabolism and detoxification, and the Nrf2 signaling pathway is intimately associated with development of atherosclerosis. During development and progression of atherosclerosis, Nrf2 signaling modulates many physiological and pathophysiological processes, like regulation of lipid homeostasis, CD36 gene expression regulation, foam cell formation, macrophage polarization, immunity regulation (Th2 differentiation and inhibition of pro-inflammatory gene expression through NFκB down-regulation), redox regulation and inflammation, improvement of endothelial dysfunction, as well as GSH synthesis and utilization (234–245). Antioxidant pathways induced by NRF2 include enzymes for the reduced GSH synthesis, utilization, and regeneration. Glutamate-cysteine ligase catalytic and modulator subunits as well as GSH synthetase are the three NRF2 targets involved in the GSH synthesis (242). The redox cycling enzymes thioredoxin, thioredoxin reductase, sulfiredoxin, peroxiredoxin, GSH peroxidase, superoxide dismutase 1, and catalase, and several GSH S-transferases, which are the enzymes mediating the elimination of ROS, are all Nrf2 targets (242). Nrf2 displays both pro- and anti-atherogenic effects in experimental animal models, and the Nrf2 pathway becomes a promising target for atherosclerosis prevention (234). Macrophage Nrf2 activates genes encoding CD36, heme oxygenase-1 and other stress proteins in response to oxLDLs and other byproducts of lipid peroxidation (240). Nrf2 depletion in macrophages leads to increased foam cell formation, increases the M1 inflammatory phenotype with enhanced expression of pro-inflammatory monocyte chemoattractant protein-1 and interleukin-6, and aggravates atherosclerosis (244, 245). Nrf2 improves endothelial function by resisting oxidative stress and mitochondrial damage, thereby delaying atherosclerosis (245); and treatment with sulforaphane, a dietary antioxidant, activates Nrf2 and suppresses p38–VCAM-1 signaling, and may provide a novel therapeutic strategy to prevent or reduce atherosclerosis (237).

### Glutathione and severe acute respiratory syndrome coronavirus 2

**FIGURE 8:**
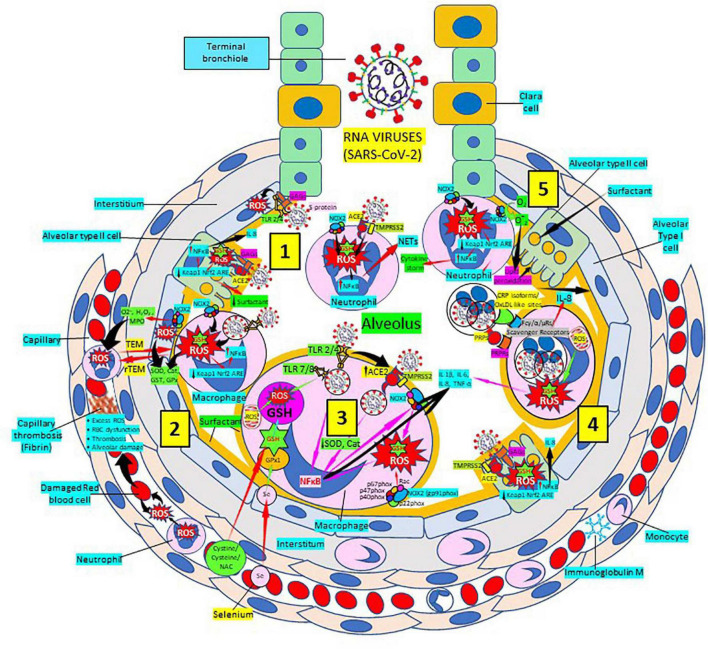
Severe acute respiratory syndrome coronavirus 2 (SARS-CoV-2) pulmonary infection, oxidative stress and antioxidant defenses. (1) After entry of SARS-CoV-2 into the alveolus, viruses invade type II alveolar cells through angiotensin-converting enzyme 2 receptors (ACE2) and glycosaminoglycans (GAGs) [see (4)], and infected cells increase reactive oxygen species (ROS) production, reduce Kelch-like ECH-associated protein 1 (Keap1)-Nuclear factor erythroid 2-related factor 2 (Nrf2)-antioxidant response element (ARE) redox regulator pathway and become defective for surfactant production. Infected cells activate nuclear factor (NF)-κB and release cytokines like interleukin (IL)-8. Alveolar type I cells augment ROS production *via* toll-like receptors (TLRs) 1 and 2. SARS-CoV-2 enhances neutrophil extracellular trap (NET) release and increases ROS production (2) SARS-CoV-2 augments macrophage’s ROS production, inhibiting Nrf2 activation and enhancing NF-κB upregulation. ROS are counterbalanced by enzymes like superoxide dismutase (SOD), catalase (Cat), glutathione S-transferase (GST), and glutathione peroxidase (GPx) to protect cells from oxidative damage caused by nicotinamide adenine-dinucleotide phosphate (NADPH) oxidase 2 (NOX2), superoxide (O_2_^–^), hydrogen peroxide (H_2_O_2_), and myeloperoxidase (MPO). Capillary neutrophils migrate to and from alveoli by *trans-*endothelial (TEM) and reverse transmigration (rTEM), respectively. SARS-CoV-2 infection can cause excessive ROS production in capillaries, red blood cell (RBC) dysfunction, thrombosis and alveolar damage. (3) Activated alveolar macrophages release increased levels of IL-1β, IL-6, IL-8, and tumor necrosis factor (TNF)-α. SARS-CoV-2-infected macrophages (*via* ACE2 and TLRs) reduce enzymes like SOD and Cat, among others, and activate NF-κB. NOX2 activation increases ROS production that enhance NF-κB activation. Glutathione (GSH) precursors (Cystine, cysteine, N-acetyl cysteine, NAC), and selenium (Se) restore GSH and GPx, respectively, to counteract the effects of ROS. (4) Alveolar macrophages engulf SARS-CoV-2-infected apoptotic cells *via* Fc (γ/α/μ) and scavenger receptors and/or pattern recognition protein receptors (PRPRs) leading to increased ROS production and cytokine release. (5) Neutrophils contribute to O_2_^–^ production, lipid peroxidation and increased oxidative stress to promote the cytokine storm. Abbreviations: TMPRSS2, Transmembrane protease Serine 2; PRPs, pattern recognition proteins. Reprinted from Labarrere and Kassab (335).

Severe acute respiratory syndrome coronavirus 2 (SARS-CoV-2) infection targets primarily the respiratory and cardiovascular systems causing COVID-19 disease identified largely by a respiratory tract infection (246, 247); sadly, many patients develop severe fatal outcomes because of overwhelming inflammation known as “cytokine storm” (248, 249), that leads to ROS-mediated cell death and tissue damage typical of RNA viruses (250). This intense inflammation is associated with damaging systemic events like oxidative stress, dysregulation of iron homeostasis, hypercoagulability and thrombus formation, acute respiratory distress syndrome, uncontrolled inflammation and organ failure (251–257) (Figure 8). Several viral infections, and the progression of virus-induced diseases, especially those associated with COVID-19, are characterized by an alteration in the intracellular redox balance (6). This imbalance disallows reactive intermediate detoxification by the cell biological systems. ROS production and associated inflammation are closely related to aging and numerous chronic diseases as diabetes, cardiovascular atherosclerosis-related diseases (144, 145) and respiratory diseases, known risk factors for developing severe illness and death in patients with SARS-CoV-2 and COVID-19 disease.

**FIGURE 9:**
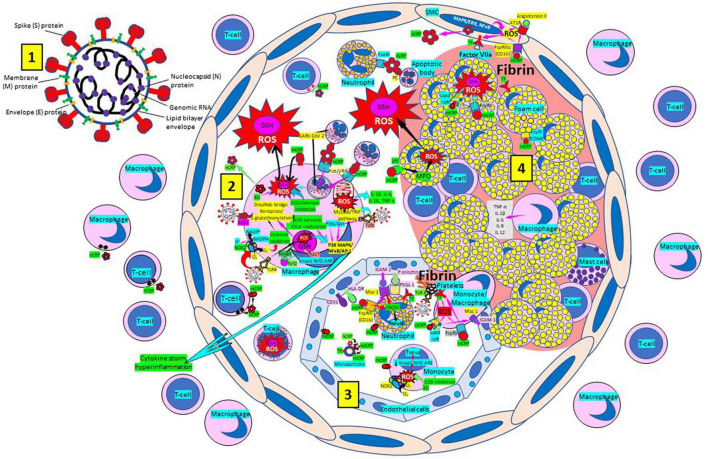
Severe acute respiratory syndrome coronavirus-2 (SARS-CoV-2) enhances oxidative stress and atherosclerosis progression. (1) SARS-CoV-2 structure. (2) SARS-CoV-2 viruses facilitate oxidative stress and inflammation in the arterial intima. Native C-reactive protein (nCRP), a marker of severe SARS-CoV-2 produced in liver, macrophages, lymphocytes, smooth muscle cells (SMC) and other cells, promotes inflammation through monomeric CRP (mCRP) enhancing intimal oxidative stress. SARS-CoV-2 binds macrophage toll-like receptor (TLR) 4 and facilitates nicotinamide adenine dinucleotide phosphate (NADP)H oxidase 2 (Nox2) activity and superoxide (O_2_^–^) production causing cysteine oxidation, disulfide bridge formation and S-glutathionylation. Xanthine oxidase (XO) and inhibition of superoxide dismutase (SOD)/catalase further facilitate O_2_^–^ cellular activity. SARS-CoV-2 can bind TLRs 2 and 4 and activate transcription factors like nuclear factor (NF)-κB facilitating cytokine storm and hyperinflammation. Excessive mitochondrial reactive oxygen species (ROS) generation further enhances cytokine production. CRP (nCRP, mCRP) can facilitate macrophage and neutrophil uptake of SARS-CoV-2-infected apoptotic cells through Fcγ and Fcα receptors, respectively (FcRs). Oxidative stress also activates the Kelch-like ECH-associated protein 1 (Keap1)-Nuclear factor erythroid 2-related factor 2 (Nrf2)-antioxidant response element (ARE) redox regulator pathway in monocytes [see (3) and macrophages (2)], releasing Nrf2 to regulate the expression of genes that control antioxidant enzymes like glutathione S-transferase (GST), facilitating glutathione (GSH) activity. Macrophages, T-lymphocytes, neutrophils and SMCs can generate mCRP increasing inflammation. (3) Monocytes, macrophages, neutrophils, endothelial cells and microparticles can generate mCRP, increase superoxide (O_2_^–^) and ROS formation and reactive nitrogen species like peroxinitrite (ONOO–), and tissue factor (TF) expression enhancing oxidation, inflammation and thrombosis. TLR 4-mediated SARS-CoV-2-binding to platelets promotes thrombosis, mCRP binding to lipid rafts and FcγRs enhances inflammation and endothelial activation allows intimal cell migration. (4) Foam cells and smooth muscle cells associated with atherosclerotic plaques enhance ROS formation, cytokine release and tissue factor (TF)-mediated fibrin deposition. Abbreviations: MAPK/ERK, mitogen-activated protein kinases/extracellular signal-regulated kinases; AT1R, angiotensin II type 1 receptor; PC, phosphorylcholine; LPC, lysophosphatidylcholine; MPO, myeloperoxidase; nnCRP, non-native CRP; TNF, tumor necrosis factor; IL, interleukin; ACE, angiotensin converting enzyme; MyD88/TRIF, myeloid differentiation primary response 88/TIR-domain-containing adapter-inducing interferon-β; PI3K/Akt: phosphatidylinositol-3-kinase/protein kinase B; MAPK, mitogen-activated protein kinase; AP-1, activator protein 1; CD31, cluster of differentiation 31; ICAM-1, intercellular adhesion molecule-1; Mac-1, macrophage-1 antigen; PSGL-1, P-selectin glycoprotein ligand-1; HLA-DR, human leukocyte antigen–DR isotype. Reprinted from Labarrere and Kassab (335).

Atherosclerosis, a chronic inflammatory disease, may be an ideal environment for the high viral replication capabilities of SARS-CoV-2 in human cells, enhancing hyper-inflammation secondary to immune system dysregulation (Figure 9) that leads to adverse outcomes, as shown in patients with cardiovascular risk factors (258, 259). In a vicious circle, feeding itself, SARS-CoV-2 may aggravate the evolution of atherosclerosis as a result of excessive and aberrant plasmatic concentration of cytokines (258–260). Atherosclerosis progression, as a chronic inflammatory mechanism, is characterized by immune system dysregulation associated with increased pro-inflammatory cytokine production, including interleukin 6 (IL-6), tumor necrosis factor-α (TNF-α), and IL-1β, as well as pattern recognition receptor proteins like CRP (170, 261–267). CRP, an active regulator of host innate immunity, is a biomarker of chronic inflammatory conditions and severe COVID-19 disease, including lung and atherosclerotic disease progression; strongly predicts the need for mechanical ventilation; and may guide intensification of treatment of COVID-19-associated uncontrolled inflammation (99, 183, 261, 262, 265, 267–269). Macrophage activation and foam cell formation may explain the elevated CRP serum levels and contribute to disease progression (Figure 9). CRP-mediated inflammation in atherosclerosis during SARS-CoV-2 infection may be related to the presence of mCRP in the lesions (188, 198, 204, 263, 267–269). The affinity of SARS-CoV-2 for ACE2 receptors makes the virus prone to cause vascular infection that could explain atherosclerosis progression and arterial and venous thrombosis (270, 271). Endothelial injury generated directly by intracellular viral replication and by ACE2 downregulation, exposing cells to angiotensin II in the absence of the modulator effects of angiotensin 1–7 (270, 271), and vascular chronic inflammation promoting the development of tissue macrophages overloaded by cholesterol (foam cells), both increase the possibility of acquiring a severe COVID-19 infection (170, 209, 258, 259, 272, 273).

### Summary and conclusions: Glutathione and early (premature) inflammaging

Chronic inflammatory diseases especially those compromising the lower respiratory system (chronic obstructive pulmonary disease, lower respiratory infections, cystic fibrosis, idiopathic pulmonary fibrosis, acute respiratory distress syndrome), diseases compromising the cardiovascular system (atherosclerosis, ischemic heart disease, stroke, and others), many other systemic inflammatory diseases like diabetes (274, 275); and actually SARS-CoV-2 causing COVID-19, all are characterized by persistent inflammation, continuous production of reactive oxygen/nitrogen species and oxidative stress that predominate over the antioxidant defenses (GSH and free radical scavenger enzymes) resulting in cell/tissue/organ aging associated with early (premature) chronic inflammation/”inflammaging.” We propose that, although inflammaging was introduced for the aging process (276–289), it could also apply to early (premature) tissue and organ aging associated with cell and tissue damage caused by excessive oxidative stress and lack of adequate antioxidant defenses, especially low GSH levels, in younger individuals. Indeed, inflammaging is associated with cell ROS over-production leading to oxidation/damage of cellular components, enhanced inflammation, and activation of cell death pathways; and oxidative stress and reduced antioxidant defenses contribute to progression of practically all diseases (290–297). In most diseases, ROS appear to have a direct connection with inflammaging and cell senescence, and oxidative stress and inflammaging increase the aging-related phenotype, and induce and aggravate the inflammatory response, creating a chronic state of systemic inflammation (290–297). Then, as proposed above, inflammaging can also be involved in aged cell/tissue processes in younger individuals. All chronic diseases, including COVID-19 with the long-COVID-19-syndrome (271), are characterized by the presence of persistent chronic inflammation and sustained generation of reactive oxygen and nitrogen species that when confronted with inadequate antioxidant defenses (likely leading components of anti-inflammaging) precipitate excessive oxidative stress. The demand for detailed analysis of the pathogenesis and clinical course of chronic diseases and viral diseases like COVID-19, as well as the use of efficacious therapies with minimal or no side effects are paramount. Here we present the antioxidant GSH as a potential unexplored way for further investigation as intervention to counteract inflammaging, premature inflammaging, inflammatory diseases and long-COVID-19-syndrome, since GSH levels are correlated with tissue and organ damage, disease severity and progression, and disease outcome (294–296, 298, 299). Enhancing GSH, mainly through NAC, GSH precursors rich in cysteine (whey protein, whey protein isolate rich in cysteine) or pro-GSH compound administration, becomes a potential treatment option for inflammatory diseases by reducing oxidative stress and cytokine expression especially in diabetic patients that also are at risk of more severe COVID-19 disease (299). GSH dysregulation might cause global immune cell autophagy decline with increased generation of proinflammatory cytokines in aging, further provoked by mitochondrial ROS signaling (293). Whey protein concentrate ameliorates lung damage and inhibits lung furin activity targeting SARS-CoV-2 S1/S2 site cleavage and SARS CoV-2 spike protein-angiotensin converting enzyme binding and could be used to protect against COVID-19 inhibiting SARS-CoV-2 cell entry (300). Glutamine, glycine, N-acetylcysteine, selenium, whey protein isolates with bonded cysteine, GSH and pro-GSH supplementation improves GSH deficiency, oxidative stress, mitochondrial dysfunction, inflammation, insulin resistance, endothelial dysfunction, genotoxicity, muscle strength, cognition and surfactant regeneration (301–307). A combination of vitamin D and L-cysteine administration significantly augmented GSH levels and lowered oxidative stress and inflammation (308, 309). Maintaining an adequate GSH redox status and 25-hydroxy-vitamin D levels will have the potential to reduce oxidative stress, enhance immunity and diminish the adverse clinical consequences of COVID-19 especially in African American communities having glucose-6-phosphate dehydrogenase (G6PD) deficiency, enzyme necessary to prevent GSH exhaustion and depletion (6, 213, 310). In normal red blood cells, pentose phosphate pathway and glycolysis are enhanced and G6PD is sufficient to produce NADPH efficiently for GSSG reduction and maintenance of GSH pool (311). G6PD-deficient cells are unable to generate enough NADPH under the condition of severe thiol depletion and GSH biosynthesis and methionine cycle are upregulated at the expense of ATP but fail to compensate for GSH depletion (311).

Severe acute respiratory syndrome coronavirus 2 can sequester mitochondria and replicate within them aging those vital organelles weakening immunity; facilitating over-stimulated or sustained inflammatory responses with interferon and cytokine release, influencing ROS production, iron storage, platelet coagulability, cytokine production stimulation, regulation of fission and fusion, mitochondrial biogenesis, and interference of apoptosis and mitophagy (312–319). By affecting all these cellular functions already impaired in aging individuals it could explain why older, comorbid patients have the most severe outcomes with COVID-19 (312) and stimulate the use of GSH and Nrf2 enhancers as well as develop new therapies to protect mitochondria. We propose that enhancement of the reduced form of GSH will reduce the body’s oxidation and inflammation associated with chronic inflammatory diseases and SARS-CoV-2 infection and COVID-19 disease (320–324). Maintaining GSH levels using therapies that do not deplete the body’s GSH (324) would be the best choice. In a patient that is overloaded with cytokine storm, the best way to fortify the immune system would be to supply it with reduced GSH, since reduced GSH is already able to provide reducing equivalents from its thiol group. This is particularly relevant when we consider GSH pathways, as well as their transcriptional regulator Nrf2, for proliferation, survival and function of T cells, B cells and macrophages (325, 326). The value of GSH and nutritional strategies like amino acids, vitamins, minerals, phytochemicals, sulforaphane to enhance cellular Nrf2, and other supplements used to restore GSH levels (327–330) as adjunct treatments for all inflammatory diseases including SARS-CoV-2 infection needs to be further emphasized. Reducing the levels of proinflammatory molecules like mCRP and nnCRP (331, 332) will further reduce the detrimental effects of inflammaging. Reestablishing the cellular metabolic homeostasis in inflammatory diseases as well as SARS-CoV-2 infection and COVID-19 disease especially in the lungs and cardiovascular system, could become paramount to balance altered innate and adaptive immunity and cell function and reduce morbimortality (333–335). Treatment of chronic inflammatory diseases and now COVID-19 appears to be complex and may resist finding a single silver bullet intervention (247) supporting the use of combination therapies (170); especially in COVID-19 bearing in mind that “no one is safe until everyone is safe” (336).

## Author contributions

CL and GK participated in the design, writing, and final corrections of the manuscript. Both authors contributed to the article and approved the submitted version.
